# The Mind After Midnight: Nocturnal Wakefulness, Behavioral Dysregulation, and Psychopathology

**DOI:** 10.3389/fnetp.2021.830338

**Published:** 2022-03-03

**Authors:** Andrew S. Tubbs, Fabian-Xosé Fernandez, Michael A. Grandner, Michael L. Perlis, Elizabeth B. Klerman

**Affiliations:** 1 Sleep and Health Research Program, Department of Psychiatry, University of Arizona College of Medicine—Tucson, Tucson, AZ, United States,; 2 Department of Psychology, Evelyn F Mcknight Brain Institute, University of Arizona, Tucson, AZ, United States,; 3 Department of Psychiatry, Perelman School of Medicine, University of Pennsylvania, Philadelphia, PA, United States,; 4 Department of Neurology, Division of Sleep Medicine, Massachusetts General Hospital, Harvard Medical School, Boston, MA, United States

**Keywords:** sleep, circadian rhythms, mental health, suicide, substance abuse, nocturnal wakefulness, psychopathology, behavioral dysregulation

## Abstract

Sufficient sleep with minimal interruption during the circadian/biological night supports daytime cognition and emotional regulation. Conversely, disrupted sleep involving significant nocturnal wakefulness leads to cognitive and behavioral dysregulation. Most studies to-date have examined how fragmented or insufficient sleep affects next-day functioning, but recent work highlights changes in cognition and behavior that occur when someone is awake during the night. This review summarizes the evidence for day-night alterations in maladaptive behaviors, including suicide, violent crime, and substance use, and examines how mood, reward processing, and executive function differ during nocturnal wakefulness. Based on this evidence, we propose the *Mind after Midnight* hypothesis in which attentional biases, negative affect, altered reward processing, and prefrontal disinhibition interact to promote behavioral dysregulation and psychiatric disorders.

## INTRODUCTION

Circadian rhythms influence human physiology and behavior, promoting wakefulness and cognition during the day and reducing cortical activity for sleep at night. Disrupted sleep increases the risk for incident and worsening psychiatric illness (Pigeon et al., 2012; Li et al., 2016; Hertenstein et al., 2019; Zhang et al., 2019; Freeman et al., 2020), and this risk may partially derive from nocturnal wakefulness, which we define as being awake during the circadian or biological night. During the biological night, cognitive capacity and mood regulation are diminished and “reason sleeps” (Perlis et al., 2016b), probably due to both intrinsic sleep loss and circadian rhythm influences. This review examines the evidence for a nocturnal propensity for dysregulated behavior, explores neurophysiological changes that may facilitate such behavior, and presents the *Mind After Midnight* hypothesis that postulates that nocturnal wakefulness-associated changes in attentional biases, affective regulation, reward processing, and executive functioning set the stage for dysregulated behaviors and psychiatric disorders.

## NOCTURNAL CHANGES IN DYSREGULATED BEHAVIOR

Nighttime is associated with an increase in impulsive and maladaptive behaviors. The empirical data for four such behaviors are considered here: suicide/self-harm, violent crime, alcohol or other substance use, and food intake.

### Suicide and Self-Harm

**FIGURE 1 |:**
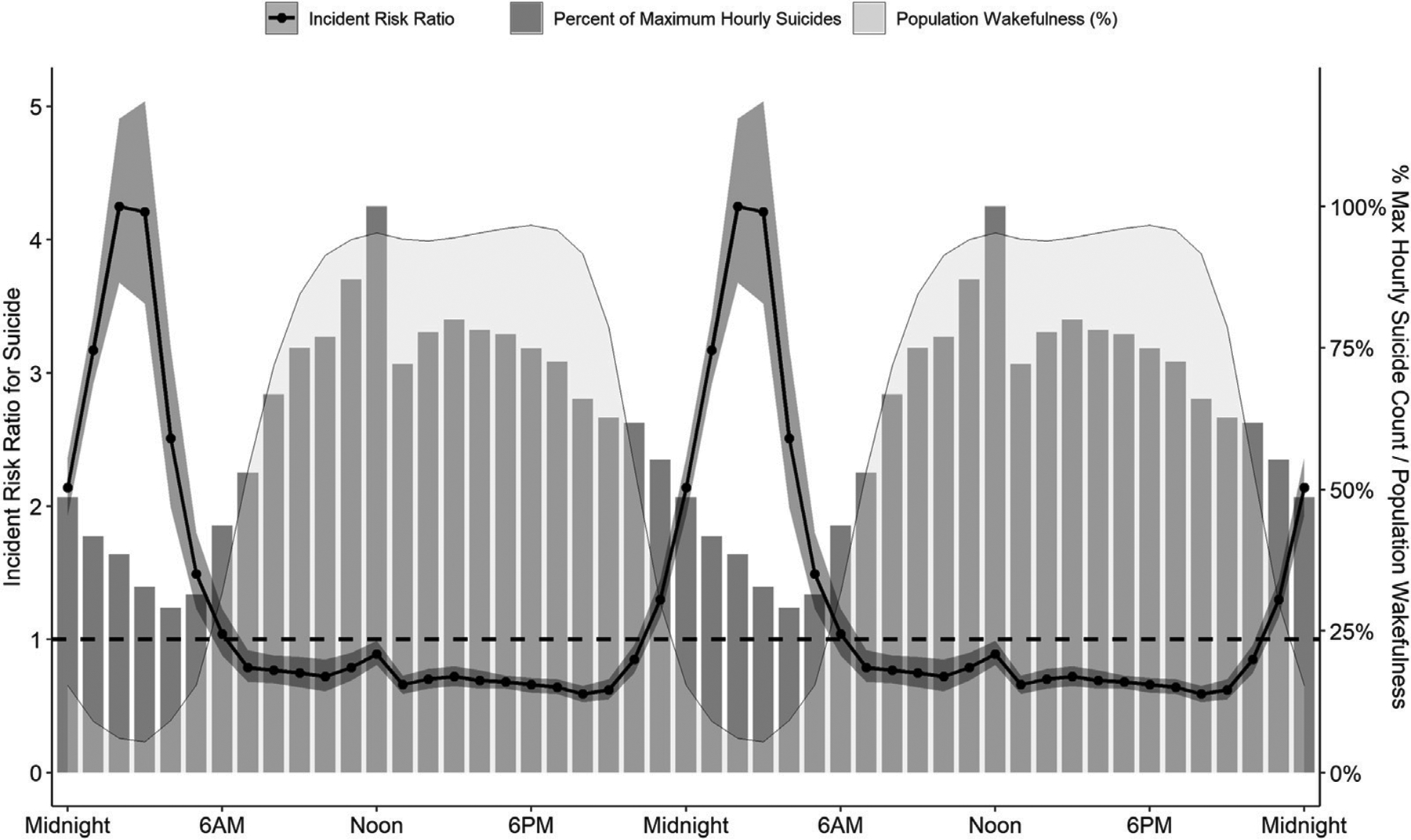
The prevalence and risk for suicide across 24 h. Although most suicides (grey bars, representing % of maximum hourly suicide count) occur around noon, population wakefulness (light grey shaded area) is also maximal at this time and throughout the afternoon and evening. After adjusting for population wakefulness, the incident risk ratio of suicide is substantially increased at night (black line, 95% confidence interval represented by dark grey band), with a 4.25-fold greater risk at 2AM than the 24-h average. Data are derived from the American Time Use Survey and the National Violent Death Reporting System for 2003 to 2010. Data are double-plotted to improve visualization of increased risk during nighttime hours.

Although most suicides occur during the day, particularly from 10 AM to 2 PM (Vollen and Watson, 1975; Barraclough, 1976; Williams and Tansella, 1987; Maldonado and Kraus, 1991; Altamura et al., 1999; Preti and Miotto, 2001; van Houwelingen and Beersma, 2001; Erazo et al., 2004), unadjusted counts are not appropriate to evaluate time-of-day differences in behavioral risk. Since wakefulness is a prerequisite to any volitional behavior, such analyses must also account for time-of-day changes in population wakefulness. In the United States, population wakefulness reaches its nadir around 3 AM, and then rises to maximal wakefulness between 10 AM and 8 PM. This means that the maximal incidence for any population-assessed behavior should be between 10 AM and 8 PM. Deviations from this pattern would reflect changes in underlying risk. For example, Perlis and colleagues (Perlis et al., 2016a) reported that the incident risk of suicide was three-fold greater between midnight and 6 AM than any other time of day (Figure 1) after adjusting for population sleep/wake timing.

Multiple subsequent analyses extend these initial findings. Veterans are eight-fold more likely to die by suicide between midnight and 3 AM (McCarthy et al., 2019), and this nocturnal peak in suicide risk does not vary by month or method of suicide (Tubbs et al., 2020b). The nocturnal propensity for suicide is apparent in some populations *without adjusting for population wakefulness*: the raw suicide counts among Japanese males over the past 40 years peaked between midnight and 3 AM (Boo et al., 2019), and the raw peak in suicides among heavy drinkers occurs around 9 PM compared to noon for non-heavy drinkers (Chakravorty et al., 2018).

Nocturnal wakefulness may also increase risk for suicidal thinking. Objectively-measured wakefulness at 4 AM predicted next-day suicidal ideation in individuals with treatment-resistant depression (Ballard et al., 2016), and self-reported wakefulness between 11 PM and 5 AM was associated with greater likelihood of suicidal ideation in both community (Tubbs et al., 2020a) and national samples (Tubbs et al., 2021). A similar pattern emerges when examining data from the Internet forum Reddit: posts on a subforum called “SuicideWatch” peaked between 2 AM and 5 AM, whereas posts on a non-suicide related subforum called “AskReddit” peaked between 8 PM and 11 PM (Dutta et al., 2021). The increase in volume of posts suggests that suicide-related thinking may be increased during those times, although the content of these posts was not analyzed.

### Violent Behavior

Homicide and violent crime also increase in prevalence at night. A 1958 study reported that murders were more likely to occur between 6 PM and 1 AM (Gibbens, 1958). Subsequent studies reported that murder rates in Manhattan peak between 6 PM and midnight (Messner and Tardiff, 1985), and that homicides in Italy peak between midnight and 6 AM (Sisti et al., 2012). More broadly, an estimated 55% of violent crimes are committed between 7 PM and 7 AM (Kelsay et al., 2017), which seems only a minor difference except that most individuals are asleep during this interval. Thus, the relative incidence is likely higher at night than during the day. A review of attempted and completed rapes (Belknap, 1987) showed that 39.5% of incidents occurred between 6 PM and midnight and 32% occurred between midnight and 6 AM. Thus, even without adjusting for population wakefulness, only 28.5% of rape incidents occur during the daytime.

### Alcohol and Other Substance Use

Illicit or inappropriate use of substances increases during the night. A comparison of daytime-only versus 24-h operation of a supervised drug consumption center revealed a peak in substance use encounters at 10 PM and a 4.7-fold greater risk of opioid overdose at night, despite the reduced overall use of heroin at that time (Montero-Moraga et al., 2020). Although circadian changes in metabolism may be responsible for this effect, this is, nonetheless, another example of how nocturnal wakefulness confers risk. Similarly, ecological momentary assessment studies have demonstrated that alcohol consumption among Latino youths is greater at night (63%) than any other time of day (Comulada et al., 2016), and that alcohol cravings in adolescents peak at 8 PM and again at 2 AM (Hisler et al., 2021). Later sleep timing in adolescents is also associated with at-risk alcohol use, binge drinking, and cannabis use (Hasler et al., 2017), possibly due to consumption during the night when the ability to drink responsibly and/or in reasonable quantities may be impaired. Finally, awakening during the night is associated with relapse to cigarette smoking (Boutou et al., 2008), perhaps due to an increased drive to smoke (withdrawal effect) and/or a reduced ability to not smoke (behavioral disinhibition).

### Food Intake

Although prolonged wakefulness and insufficient sleep enhance metabolic demand with a concomitant increase in food intake, eating at night is not solely a consequence of metabolic demand. At night, food choices are skewed toward carbohydrates, lipids, and processed foods, and away from fruits and vegetables, with total intake often exceeding the caloric demands of prolonged wakefulness (Knutson, 2012; Markwald et al., 2013; Shechter et al., 2014). These changes might reflect impairments in metabolic decision-making and/or an increased focus on hedonic reward (Broussard and Van Cauter, 2016). One extreme example is people with night-eating syndrome, who consume the majority of their calories during uninhibited food intake at night (Vander Wal, 2012). Although not well understood, night eating syndrome is hypothesized to involve changes in reward processing and serotonergic signaling (Stunkard et al., 2009; Mendoza, 2018), all of which result from and contribute to inappropriate wakefulness at night. The possibility that nocturnal changes in reward circuitry motivate changes in food-related behavior is also seen in animal models; in mice, for example, homeostatic intake of food peaks during the nighttime active phase (the equivalent of the human day) while hedonic food intake peaks during the daytime rest phase (the equivalent of the human night) owing to changes in dopaminergic neurons (Koch et al., 2020).

## NOCTURNAL CHANGES IN THE BRAIN

Increased nocturnal risk for maladaptive behaviors likely results from sleep- and circadian-changes in neurophysiology during nocturnal wakefulness. Note that for this purpose, nocturnal wakefulness does not include short (e.g., <3 min) awakenings from sleep episodes. The following section discusses synaptic saturation, neurotransmitter activity, affect regulation, reward processing, and executive function as potential mechanisms of dysregulated nocturnal behaviors.

### Synapses and Neurotransmitters

Under the synaptic homeostasis hypothesis (Tononi and Cirelli, 2019), continuous wakefulness leads to synaptic saturation and impaired signal transmission. Much as a muscle fiber in continuous contraction loses strength, the saturated central nervous system eventually loses its capacity for effective cortical activity and cognition. Ordinarily, the sleep and circadian systems overlap at night to restore synaptic homeostasis via synaptic downscaling and reduced cortical excitability (Ly et al., 2016). By contrast, wakefulness at night provokes cortical activity at a time when synapses are saturated and cortical responses are impaired.

Nocturnal wakefulness also involves circadian changes in neurotransmitter availability and signaling. Although norepinephrine and serotonin decrease at night in anticipation of sleep, dopamine peaks during the latter half of the dark period in both (diurnally active) humans (Doran et al., 1990) and (nocturnally active) rats (Castaneda et al., 2004), indicating it is tied to the rhythms of light and dark, not sleep and wake. This circadian rhythm in dopamine plays an important role in the regulation of sleep (particularly REM sleep) (Monti and Monti, 2007; Kashiwagi et al., 2021), but may have unintended consequences when coincident with nocturnal wakefulness (Alonso et al., 2021). Human studies show a reduction in available dopamine D2 receptors (inhibitory) in the ventral striatum following sleep deprivation (Volkow et al., 2008; Volkow et al., 2012), which suggests an increase in dopaminergic activation. This may not only extend wakefulness but also adversely affect psychiatric symptoms associated with dopaminergic dysregulation (e.g., sensation seeking, impulsivity, delusional thinking). Nocturnal wakefulness may also produce a stress response with a surge in adrenergic signaling that further weakens prefrontal cortical activity, increases limbic/subcortical responsivity, and increases reflexive, impulsive decision-making (Arnsten, 2015; Woo et al., 2021).

### Positive and Negative Affect

Positive and negative affect exhibit distinct circadian rhythmicity. Positive affect peaks during the day and reaches its nadir during the night (1 AM to 4 AM) (Murray et al., 2002; Murray et al., 2009; Golder and Macy, 2011; Hasler et al., 2012; Miller et al., 2015; Emens et al., 2020). Conversely, negative affect remains steady throughout the day before peaking sharply during the nighttime trough of positive affect (Wirz-Justice, 2008; Kline et al., 2010; Golder and Macy, 2011; Dzogang et al., 2017; Emens et al., 2020; Ten Thij et al., 2020). Nocturnal wakefulness thus occurs at a time when positive affect is lowest and negative affect highest, which shapes how an individual attends to and interprets information. In-lab circadian investigations have shown that wakefulness during the habitual sleep period is linked to significantly worse subjective ratings of mood (Boivin et al., 1997; Chellappa et al., 2020). Nocturnal wakefulness also impairs regulation of negative affect (Harrington et al., 2021) and promotes depressive, anxious, and/or paranoid thinking (Kahn-Greene et al., 2007; Killgore, 2010; Reeve et al., 2015; Sheaves et al., 2016; Cosgrave et al., 2018; Reeve et al., 2018. Nocturnal wakefulness can also increase exposure to artificial light that can further disrupt sleep-wake rhythms and potentially tilt the balance of affect towards negative mood (Bedrosian and Nelson, 2017).

### Reward Processing

Reward is hypothesized to motivate behaviors that maximize survival. The survival benefit of many behaviors, however, varies as a function of time of day: for a diurnal animal, foraging for food may be beneficial during the day when food is visible, but dangerous at night due to predation. Preliminary evidence shows that activity in the nucleus accumbens, caudate, and putamen varies in accordance with the day/night cycle (Byrne et al., 2019). In one study, the responses to high- and low-calorie foods in the putamen and ventral striatum were significantly reduced in the evening, even as subjective preoccupations about food were elevated (Masterson et al., 2016). A comparison of striatal responses to a monetary reward task demonstrated that 1) reward activation was greatest in the afternoon (Hasler et al., 2014), and 2) striatal activation during reward was significantly reduced during circadian misalignment (Hasler et al., 2021). Thus, subcortical processing of reward appears to vary as a function of time of day.

Sleep deprivation adds another layer of complexity to reward processing as total sleep deprivation is known to increase risky decision-making (Womack et al., 2013). Rewards following sleep loss are accompanied by greater activation of the ventral striatum (Gujar et al., 2011) and reduced activity in the anterior insula (Venkatraman et al., 2007; Venkatraman et al., 2011), possibly reflecting an exaggerated expectation of reward alongside diminished sensitivity to negative consequences. Interestingly, elevated activation in the ventral striatum at baseline predicts greater caloric and carbohydrate intake during the latter part of sleep deprivation (Satterfield et al., 2018). This suggests that individuals with elevated baseline reward sensitivity are more likely to act impulsively (e.g., overeat) during sleep deprivation because of acute increases in activity in the nucleus accumbens. No studies to date have examined interactions between sleep deprivation and circadian timing on reward processing.

### Executive Function

The prefrontal cortex integrates sensory input along with assessments of subcortical salience to manage adaptive behavioral responses related to long-term planning, risk-assessment, behavioral inhibition, and cognitive control (collectively referred to as executive function (Diamond, 2013)). Prefrontal performance is particularly sensitive to accumulating sleep debt as indexed by increased sleep delta activity (0.5–4.0 Hz on EEG), and enforced sleep deprivation results in maximal accumulation of delta activity over the prefrontal cortex relative to other brain areas (Cajochen et al., 1999; Munch et al., 2004). Waking midline theta activity (4–8 Hz) arising from the anterior cingulate cortex also increases as a function of sustained wakefulness (Cajochen et al., 1995; Cajochen et al., 1999; Finelli et al., 2000; Cavanagh and Frank, 2014). Even under protocols that include wakefulness during the biological night without sleep deprivation (i.e., forced desynchrony protocols), delta and waking theta frequency power in the EEG during sleep measured at night are greatest over the prefrontal region, which suggests that the rate at which wakefulness incurs sleep-pressure/cortical-fatigue is accelerated at night versus the day (Cajochen et al., 1999; Finelli et al., 2000; Cajochen et al., 2002; Niggemyer et al., 2004; Munch et al., 2007). In addition, insufficient sleep and circadian changes associated with the biological night are linked to reduced cortical excitability (Ly et al., 2016), reduced functional connectivity within the prefrontal cortex (Verweij et al., 2014), and altered connectivity between the prefrontal cortex and the amygdala (Killgore, 2013; Muto et al., 2016).

This frontal cortical dysfunction may explain the profound deficits in executive function observed during nocturnal wakefulness and/or following sleep deprivation (Nilsson et al., 2005; Kuula et al., 2018; Cross et al., 2021). Cognitive functions such as working memory, complex attention, and problem solving deteriorate (May and Kline, 1987; Libedinsky et al., 2013), leading to an increase in attentional lapses, performance errors, and injuries (May and Kline, 1987; Nelson et al., 1995; Wright et al., 2012). Response inhibition is diminished (Drummond et al., 2006), decision-making becomes inflexible (Wimmer et al., 1992; Harrison and Horne, 1999; Maddox et al., 2009; Slama et al., 2018), and impaired risk management manifests in risky, net-loss decision-making (Killgore et al., 2006; McKenna et al., 2007; Venkatraman et al., 2007; Pace-Schott et al., 2012; Womack et al., 2013; Mantua et al., 2021). Behavioral inhibition, which is maximized during the circadian day (May and Hasher, 1998), declines as wakefulness proceeds into the circadian night (Hasler et al., 2021). As an additional caveat, executive performance is significantly disrupted immediately upon awakening, a phenomenon known as sleep inertia. Cognitive deficits associated with sleep inertia are exacerbated by chronic short sleep and abrupt awakenings during the circadian night (Horne and Moseley, 2011; McHill et al., 2019), suggesting that both sleep loss and circadian factors contribute to nocturnal deficits in executive function. Of note, some of these studies were performed in military, physician, or other participants who were highly skilled and motivated for the task (May and Kline, 1987; Nelson et al., 1995; Harrison and Horne, 1999; Maddox et al., 2009; Horne and Moseley, 2011; Mantua et al., 2021). Unfortunately, in all these studies, assessment was done during the day, not during the night of sleep deprivation.

## THE MIND AFTER MIDNIGHT

**FIGURE 2 |:**
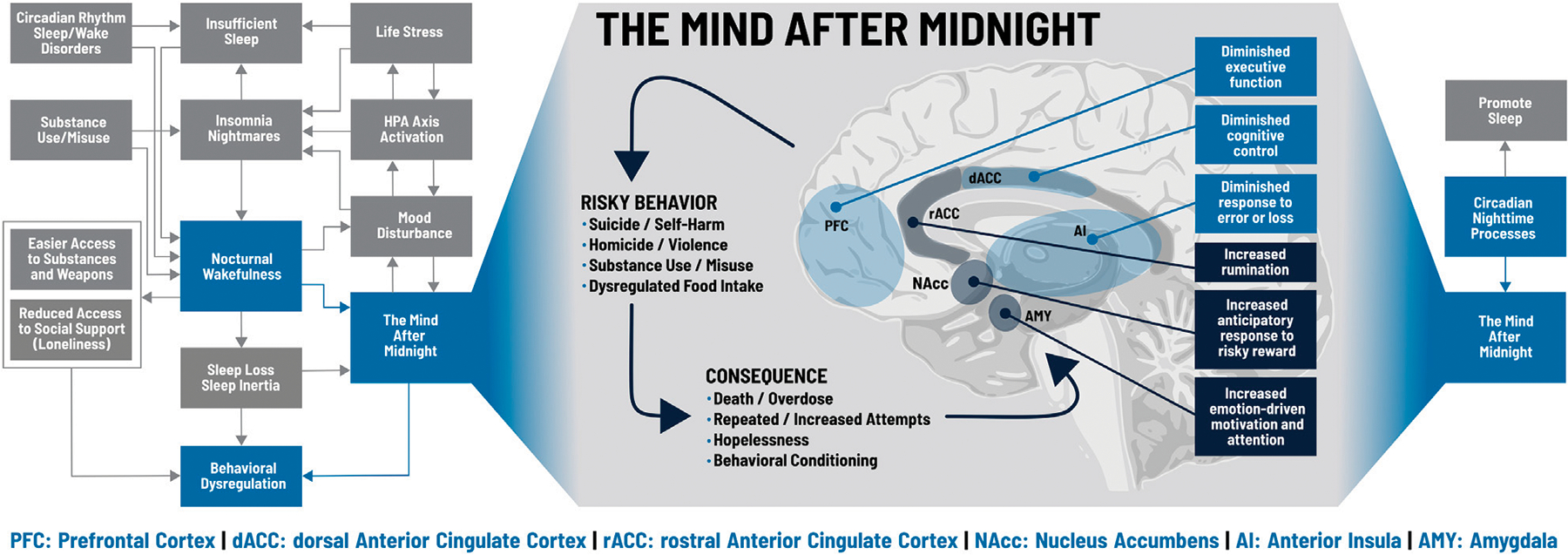
The Mind after Midnight. Blue boxes on the left and right sides indicate key processes within the model discussed in the text; grey boxes indicate additional components not specifically addressed. During nocturnal wakefulness, upregulation of the amygdala (AMY), nucleus accumbens (NAcc), and the rostral anterior cingulate cortex (rACC) increase emotionally driven salience, attention, and motivation, skew anticipation of risky rewards, and drive excess rumination. Conversely, impairments in the prefrontal cortex (PFC), dorsal anterior cingulate cortex (dACC) and anterior insula (AI) lead to executive dysfunction, diminished cognitive control, and insensitivity to error or loss. These changes promote a cycle of risky behaviors and consequences that can spiral out of control. Figure adapted from Perlis et al. (2016b).

“Is it a bad thing to be awake when reason sleeps?” (Perlis et al., 2016b). Sleep loss- and circadian-dependent changes in molecular signaling, affect, reward processing, and executive function provide conceptual support for behavioral dysregulation, and empirical studies demonstrate a pattern of increased risk with nocturnal wakefulness. The reasons for being awake at night can also contribute to behavioral dysregulation: insomnia, nightmares, short sleep, or circadian rhythm disorders can produce nocturnal wakefulness, hypervigilance, and limited emotion regulation; hypnotics, alcohol, and other substances can produce rebound wakefulness, even if intoxication remains; and stress, anxiety, and mood disturbances promote nocturnal wakefulness even as they undermine decision-making. Figure 2 illustrates how biological and psychological factors intersect at nocturnal wakefulness and lead to *The Mind After Midnight* hypothesis, which we propose as a framework for interpreting current findings and a guide for future research and interventions, including therapy.

### The Hypothesis

In brief, the circadian influence on (neuro)physiology differs over the 24 hours. During the day, molecular levels, neuronal activities and/or responsivity are tuned to our usual behavior, wake, which includes locomotor activity, eating, conscious interactions with our environment/people. During the night, these parameters are tuned to the usual behavior of sleep. So, if we are awake at these times, neurophysiology is prone to foster behavioral dysregulation, especially when these time-of-day effects are combined with sleep loss or disruption. We hypothesize that nocturnal wakefulness produces the Mind after Midnight, a combination of circadian-dependent decreases in molecular and cortical activity and responsiveness with sleep-loss-dependent alterations in synaptic signaling and cortical connectivity. Heightened negative affect and diminished positive affect produce a narrowed attentional focus on neutral or negative stimuli which are assigned incorrect or excessive emotional salience by an overactive amygdala. This biased information then feeds into an aberrant reward/motivation system characterized by elevated dopamine levels, altered dopamine receptor availability, increased activity in the nucleus accumbens (ventral striatum), and reduced activity in the caudate and putamen (dorsal striatum), which results in diminished activity during reward receipt but maximized anticipation/expectation of reward, particularly for risky behaviors.

This combination of negative attentional focus, skewed reward processing, and motivational impulsivity then rises to the prefrontal and anterior cingulate cortices. Ordinarily, coordinated activity between the dorsal anterior cingulate cortex (dACC), the rostral anterior cingulate cortex (rACC), and various regions of the prefrontal cortex (PFC) would exert cognitive control to suppress negative emotional distractors and focus on goal-oriented behavior (Etkin et al., 2006; Buckner et al., 2008; Hamilton et al., 2011; Diener et al., 2012; Niendam et al., 2012; Shenhav et al., 2016; Berry et al., 2017). Sleep loss and nighttime circadian phase, however, are associated with synaptic saturation and cortical fatigue that disrupt this coordination and produce ruminative, self-referential thinking characterized by fear/anxiety, depression, and hopelessness (Broyd et al., 2009; Sheline et al., 2009; Yoshimura et al., 2010; Toyoda et al., 2011; Nejad et al., 2013; Kaiser et al., 2015). Long-term planning and behavioral inhibition are diminished in favor of high-risk decision-making and cognitive inflexibility, leading to repeated, maladaptive behaviors that do not respond to negative feedback.

These conditions make it clear how poor mood, impaired judgement, and impulsivity could lead to maladaptive behaviors and catastrophic outcomes. For example, a previously abstinent heroin user who successfully manages cravings during the day may experience greater cravings and diminished resistance at night. The appeal of heroin use becomes more desirable and satisfying than the potential costs, and a single impulsive decision leads to a relapse. Further, a dose that was sufficiently rewarding during the day may be insufficient at night, potentially resulting in an increased dose, repeated use, or both, as the risk of overdose is downplayed or discarded. If this process repeats night after night, a conditioned pattern of nighttime heroin use may emerge (Gillman et al., 2019).

Another example is a college student experiencing nocturnal wakefulness due to a delayed sleep schedule and insomnia. Negative mood is at its peak, and the student feels isolated and alone, leading to endless rumination on prior negative relationship experiences. These particular experiences quickly, and inappropriately, generalize to all relationships, thus creating a sense of hopelessness and despair. Suicide, previously inconceivable, emerges as an escape from loneliness and pain, and before the costs of suicide are considered the student has acquired the means and is prepared to act at a time when no one is awake to stop them.

These examples demonstrate how negative affect, impaired judgment, and impulsivity during nocturnal wakefulness may increase the incidence and appeal of dangerous ideas, and promote dysregulated behaviors.

### Future Directions

The *Mind after Midnight* hypothesis conceptualizes how disrupted or decreased sleep could contribute to psychiatric disorders and unsafe behaviors, and offers a unique opportunity for meaningful intervention. The *Mind after Midnight* hypothesis also integrates well with psychological models of acute behavioral risk, such as the Suicide Crisis Syndrome or Acute Suicidal Affective Disturbance (Schuck et al., 2019; Voros et al., 2021).

All aspects of this hypothesis require empirical validation. We have reviewed reports of data collected during the biological night, and, when such data were not available, we used reports of data from after a night of sleep loss. To test the *Mind after Midnight* hypothesis, data need to be collected during the biological night (including - when possible - using protocols that do not cause sleep loss). Adding time-of-day as a relevant metric to all studies is also extremely important, as measuring time-of-day (and time since awakening) effects on a variety of outcomes could identify behaviors (or individuals) that are most vulnerable or resilient to nocturnal wakefulness. Mechanistic studies could use known electrophysiological measures of cognition, emotion, and cortical activity (e.g., frontal alpha asymmetry, frontal midline theta, fMRI) to measure nocturnal trait and state changes in activity. Future work could also distinguish the effects of prolonged wakefulness from abrupt nighttime awakenings and the distinctions between sleep loss and circadian processes.

The *Mind after Midnight* naturally lends itself to clinical applications. The simplest solution would be to help vulnerable individuals to sleep through the night, thus reducing their exposure to the times with elevated risk due to behavioral dysregulation. Example interventions would include treatment for insomnia, pain, cravings, or other causes of nighttime awakening, changes in the environment, and/or the provision of greater social supports when individuals are awake at night.
